# *Bacillus subtilis* CF-3 Volatile Organic Compounds Inhibit *Monilinia fructicola* Growth in Peach Fruit

**DOI:** 10.3389/fmicb.2019.01804

**Published:** 2019-08-07

**Authors:** Minshun Zhou, Peizhong Li, Shiyuan Wu, Pengyu Zhao, Haiyan Gao

**Affiliations:** ^1^School of Life Sciences, Shanghai University, Shanghai, China; ^2^Shanghai Key Laboratory of Bio-Energy Crops, Shanghai, China

**Keywords:** *Bacillus subtilis* CF-3, volatile organic compounds, *Monilinia fructicola*, biological control, antifungal effect

## Abstract

In this study, we evaluated the effects of volatile organic compounds (VOCs) produced by *Bacillus subtilis* CF-3 in inhibiting *Monilinia fructicola in vitro* and *in vivo*. In the *in vitro* experiments, the effect of VOCs on the growth of the pathogenic fungi was explored by using plate enthalpy test; mycelial morphology was studied by scanning electron and transmission electron microscopy; and fatty acid contents in the cell membrane were assessed by gas chromatography-mass spectrometry (GC-MS). The results indicated that treatment with benzothiazole and CF-3 for 24 h, in the form of a fermentation broth (24hFB), significantly inhibited the germination of fungal spores, modified hyphal and cell morphology, and decreased the cell membrane fluidity and integrity. In the *in vivo* experiments, the effect of VOCs on the defense mechanism of peach fruit toward *M. fructicola* was studied, and we found that benzothiazole and CF-3 24hFB inhibited the activity of the pathogenic enzymes (pectinase, cellulase) secreted by *M. fructicola* to reduce the decomposition of plant tissues, activate the antioxidant enzymes (peroxidase, polyphenol oxidase, catalase, and superoxide dismutase) in the fruit to eliminate excessive reactive oxygen species in order to reduce plant cell damage, and trigger the disease-resistant enzymes (phenylalanine ammonia-lyase, chitinases, and β-1,3-glucanase) to enhance the resistance of peach fruit to *M. fructicola* and inhibit its growth. This study suggests that CF-3 VOCs could activate disease-resistant enzymes to prevent the invasion of pathogenic fungi and induce resistance in peach, thereby providing a theoretical basis for future applications.

## Introduction

Due to the inadequate treatment in each production step, *Monilinia fructicola* infested on the peach fruit causes decay, resulting in large economic losses worldwide (Liu et al., 2008; Hu et al., 2011). Previously, postharvest fruit diseases were controlled *via* refrigeration or modified atmosphere storage and fungicides (Zheng et al., 2013). Physical methods may not provide efficient inhibition, whereas chemical methods may cause food safety and environmental pollution issues (Schreinemachers and Tipraqsa, 2012). Thus, *Bacillus subtilis* is widely studied for fruit preservation due to its rich physiological characteristics and strong environmental adaptability, which has broad application prospects in the control of plant diseases as an extremely important bio-control resource (Ongena et al., 2005).

In recent years, the antifungal effect of *B. subtilis* has gained immense attention in research. *B. subtilis* inhibits various plant pathogens and has an antagonistic effect on several plant fungi diseases that affect the plant roots, branches, flowers, and fruits (Senthil et al., 2011; Gava et al., 2019). In the existing report, *B. subtilis* antagonizes more than 30 types of plant pathogens (Moyne et al., 2004). It is widely distributed in nature and is nontoxic and harmless to humans and children, easy to isolate and cultivate, and has broad-spectrum antifungal activity and strong antireverse ability to produce antibiotics and enzymes such as peptides, lipopeptides, polyenes, and amino acids (Ahimou and Deleu, 1999), thus being extremely beneficial in biological control and fruit preservation.

Moreover, recent studies reported that *B. subtilis* can produce not only antibiotics and antifungal proteins (Zhang et al., 2019) but also a series of VOCs (Leelasuphakul et al., 2008), which can effectively inhibit the growth of postharvest pathogens of fruits and vegetables. Waewthongrak et al. (2015) reported that the cyclolipopeptides secreted by *B. subtilis* ABS-S14 had a broad-spectrum antifungal effect, thereby reducing the *Penicillium* strains exposed to *B. subtilis* and cell-free culture by 96.2 and 90.9%, respectively. Ambrico and Trupo, (2017) further confirmed that Iturin A produced by *B. subtilis* ET-1 can effectively inhibit the occurrence of blue and gray molds on lemon and strawberry fruits, respectively.

Compared with the nonvolatile antifungal substances, VOCs are more likely to exert antifungal activity *via* air and soil. During preservation, the VOCs produced by *B. subtilis* can be combined with the modified atmosphere storage in order to achieve anticorrosion. Although *B. subtilis* VOCs are proved to be effective in inhibiting the growth of pathogens (Vespermann et al., 2007), the mechanism of VOCs remains unclear. It was reported that *B. subtilis* VOCs could destroy the mycelial morphology, exhibiting a strong antimicrobial effect (Zhou et al., 2011), and could prevent the pigment production in pathogenic bacteria (inhibition rate 43–93%; Liu et al., 2008).

Peaches are favorably cultivated in China, and any decay caused by *M. fructicola* may result in more than 20% yield loss (Zhang et al., 2015a,b). In the previous study, we isolated and identified the *B. subtilis* CF-3 (Gao et al., 2016) and total 74 potential VOCs in the fermentation process (Gao et al., 2017). And the main VOCs were noted as 1-octanol, benzaldehyde, 3-methylbutanal, benzoic acid, methoxy-phenyl-oxime, anisole, benzothiazole, and 2,4-di-tert-butylthiophenol, which have different antimicrobial effects (Gao et al., 2018). Among them, benzothiazole, a crucial compound that inhibited *M. fructicola* was assessed. In order to clarify the mode of VOC action both *in vivo* and *in vitro*, this article highlights the effect of VOCs in inducing fruit resistance against *M. fructicola* and attempts to explain how VOCs inhibit pathogen, thereby laying the foundation for future applications.

## Materials and Methods

### Preparation of Strain and 24hFB

*B. subtilis* CF-3 was isolated from fermented bean curd and identified by the Laboratory of Food Safety and Quality Control (School of Life Sciences, Shanghai University), which was registered in the China Center for Type Culture Collection, CCTCC M 2016125 (Gao et al., 2016). *B. subtilis* CF-3 was cultivated for 7 days at 37°C, using LB solid medium (Gao et al., 2017).

*M. fructicola* (provided by the Beijing University of Agriculture) was cultivated for 7 days at 25°C, using PDA solid medium (Gao et al., 2017).

To obtain 24hFB, *B. subtilis* CF-3 was cultivated on LB solid medium for 24 h at 37°C, then gently scraped with an inoculating loop and then transferred into 100 ml LB liquid medium in a conical flask and cultivated at 37°C in a rotary shaker at 150 rpm for 24 h, and was eventually diluted to a concentration of about 10^8^ cfu/ml through a hemocytometer. Moreover, a fumigation treatment was performed.

### Fumigation Treatment by *B. subtilis* CF-3 Volatile Organic Compounds

The method described by Arrebola et al. (2010) and Jiang et al. (2014) was used to evaluate the inhibitory effects of VOCs produced by *B. subtilis* CF-3 against *M. fructicola*. About 20 μl of *B. subtilis* CF-3 fermentation broth after 24 h fermentation was aspirated and evenly spread on LB solid medium. Subsequently, a plug (Ø7 mm) from the agar of the fungus, which was incubated for 7 days, was punched and placed at the center of a fresh PDA medium. Eventually, the LB solid medium was inverted on the fungus-attached PDA solid medium and sealed with a parafilm to reduce the loss of VOCs. The sealed petri dish was placed in an electrothermal incubator at 28°C for 7 days.

### Effects of Volatile Organic Compounds on *M. fructicola* Spore Germination

Preparation of pathogenic spore suspension: 5 ml of sterile water containing 0.05% Tween 80 was added to the PDA plate of *M. fructicola* precultivated for 7 days, and the solution was then filtered through eight layers of sterile cheesecloth, and then, the concentration was adjusted to 1 × 10^4^ conidia/ml using distilled water.

The treatment settings were as follows: blank control group (no treatment, distilled water); positive control group (500 mg/L thymol solution with distilled water); 1 mol/L benzothiazole; and CF-3 24hFB.

The treatment solution was evenly spread on LB solid medium, and the spore suspension (20 μl) was evenly spread on fresh PDA medium. The reagent-coated medium was inverted on the PDA coated medium with spore suspension and was further sealed with a parafilm and placed in a 28°C incubator for cultivation. Simultaneously, LB solid medium coated with the same volume of distilled water was used as a blank control, and the equal amount of thymol solution was added as a positive control, followed by cultivation under similar conditions. Set up three parallels for each processing group. The germination of spores was, respectively, observed by light microscopy at 2, 4, 6, 8, 10, and 12 h, and the length of the germ tube exceeded half of the maximum diameter of spores as the spore germination standard. When the spore germination rate in the blank control group exceeded 90%, the rate of all treatment groups was measured and calculated. The formula for calculating the spore germination rate is as follows:

$$R_{1}\;=\;\frac{n}{t}\;\times \;100\%$$

where *R*_1_ is the spore germination rate; *n* is the number of spores that have sprouted; and *t* is the total number of spores.

### Effects of Volatile Organic Compounds on *M. fructicola* Morphology

Preparation of electron microscope fixative (2.5% neutral glutaraldehyde): 10 ml glutaraldehyde solution (25%), 50 ml phosphate buffer solution (0.2 M, 0.588 g NaH_2_PO_4_·2H_2_O, 5.8 g Na_2_HPO_4_·12 H_2_O, dissolved and mixed in 100 ml sterile distilled water, and adjust the pH to 7.2–7.4, standby), and 40 ml sterile distilled water. After mixing, a 2.5% neutral glutaraldehyde solution was prepared.

The scraped hyphae (blank control, single VOC treatment, CF-3 24hFB) were soaked in electron microscope fixative and sent to Shanghai Yuyi Testing Center for electron microscopy.

### Effect of Volatile Organic Compounds on the Fatty Acid Content of *M. fructicola* Cell Membrane

The accurately weighed 0.2 g of the fumigated fungal sample was added in a round bottom flask, with an addition of 100 ml of methanol to reflux for 2 h, and the reflux was steamed 5 ml with a rotary evaporator.

The extracted lipid concentrate was hydrolyzed with potassium hydroxide-methanol solution (11 g/L) at 90°C for 10 min. Total fatty acids were then derived with 2 ml sulfuric acid methanol (10%, V/V) at 90°C for 20 min. After adding 250 μl of the internal standard (1 g/L methyl nonanoate), the fatty acid solution was extracted with 2 × 6 ml of isooctane and occasionally oscillated. The water in the isooctane layer was removed with anhydrous sodium sulfate. Eventually, it was concentrated by rotary evaporation to 1 ml and stored at 20°C for analysis.

According to Tang et al. (2011), an 1 μl sample was injected in HP-5 ms capillary column, and the fatty acid content was analyzed using GC-MS. All samples were analyzed in triplicate, and the standard deviation was calculated.

### Effects of Volatile Organic Compounds on the Content of Ergosterol in the *M. fructicola* Cell Wall

Accurately weighed 0.2 g of the sample was placed in a 40 ml covered vial. About 20 ml NaOH solution (5 g NaOH, 5 ml sterile distilled water, and 95 ml methanol) and 20 μl 1 mg/ml internal standard stock solution [accurately weighed 7-dehydrocholesterol standard (VD3) 100 mg, corrected to 0.1 mg, dissolved in methanol, and diluted to 100 ml, to get 1 mg/ml internal standard stock solution] were hydrolyzed at 80°C for 2 h and then cooled at room temperature. From the above hydrolyzate, 5 ml solution was added in a 15 ml stoppered tube and added 3 ml of n-hexane, and the hydrolyzate was vortexed for 2 min. Next, 1 ml of the supernatant was added into a chromatography bottle, and then, the solvent was dried in a 40°C water bath, and to this 300 μl BSTFA was added at 80°C for 45 min and analyzed by GC-MS after cooling.

According to Saraf et al. (1997), Pasanen et al. (1999), Sha et al. (2008), and Liu et al. (2007), the qualitative analysis was accomplished in selected ion monitoring (SIM) mode with *m*/*z* 363 for ergosterol and *m*/*z* 351 for internal standard. All samples were analyzed in triplicate, and the standard deviation was calculated.

About 1 ml of ergosterol standard solution with a concentration of 5, 10, 20, 50, and 100 μg/ml, respectively, and 100 μl of 1 mg/ml internal standard stock solution were added in a chromatography bottle. After evaporation of the solvent in a 40°C water bath, 300 μl of BSTFA was added and derived at 80°C for 45 min. The standard series of ergosterol solutions were determined and analyzed by GC-MS in similar conditions. The peak area was analyzed by regression analysis to obtain the integrated peak area of ergosterol and internal standard. The integrated peak area and internal standard of ergosterol were obtained. The ratio of the integrated peak area of ergosterol to the internal standard peak area was used as the ordinate (Y), and the ratio of the ergosterol mass concentration to the internal standard mass concentration was used as the abscissa (X) in order to establish a standard curve of ergosterol.

### Effects of CF-3 Volatile Organic Compounds on Related Enzyme Activities and Indicators in Peach Fruit

Peach fruit is the “Zhaohui” variety of Shandong Mengyin, which was purchased from Shanghai Fruit Wholesale Market. We chose fruits that were disease-free, had no obvious wounds, had a neat appearance, and had similar maturity and size. Next, they were pruned and immersed in a 0.1% sodium hypochlorite solution for 1 min and then rinsed with tap water and air-dried for further use.

We took the peach fruit, which was disinfected and air-dried, used a sterilized hole punch with a diameter of 7 mm to make a hole with a diameter of 7 × 5 mm in the center of the largest section of the peach fruit, injected 20 μl of the prepared fungal spore suspension with a syringe of 1 ml, and dried it until further use. Next, we kept 10 peaches on a plastic tray that was sterilized with 75% ethanol (4 × 3 rows of grooves), by placing 1 peach in each groove, and then placed two pieces of filter paper in the remaining two grooves separately (90 mm diameter). Subsequently, 200 μl of reagent or CF-3 24hFB was added to each of the two filter paper sheets (benzothiazole 1 mol/L). Then, it was quickly added into a prepurchased cardboard box (45 cm × 35 cm × 10 cm) and was sealed; the box was wrapped in a gas-conditioned bag and placed at room temperature (25°C). Samples were unpacked every day from Day 1 for a total of 4 days, with three replicates in each treatment. The disease speckle diameters were measured, and the inhibition rate was calculated to contrast treatment effects. Due to the instability of VOCs, they were discarded directly after unpacking and were not resealed.

The peach fruit treatment settings are as follows: (1) blank control group (no treatment); (2) 1 mol/L benzothiazole; and (3) CF-3 24hFB.

We randomly selected 15 peaches from three boxes, five from each box. Namely, there were 15 peaches removed each day for 4 days per treatment. The reduction of the fungal infection *in vivo* was assessed by inhibition rate, and the formula is as follows:

$$R_{2}\;=\;\frac{d_{0}-d}{d_{0}}\;\times \;100\%$$

where *R*_2_ is the inhibition rate; *d*_0_ is the disease speckle diameter of blank control group; and *d* is the diameter of treatment group.

The sampling was done by cutting off the outermost 1 cm pulp of the punching part and cutting the 0.5 cm deep pulp at the punching part. The 0.5 cm deep pulp was separated into small pieces, frozen in liquid nitrogen, and stored in a refrigerator at −80°C for further use.

#### Measurement of Polygalacturonase and Cellulose

To extract polygalacturonase (PG) and cellulose, 10.0 g peach fruit sample was ground in 95% ethanol in an ice bath and centrifuged at 4°C and 12,000 × *g* for 20 min. Then, it was added in 80% ethanol and centrifuged at 4°C and 12,000 × *g* for 20 min. Extraction buffer solution was added to the precipitate. After centrifugation, the supernatant was collected and stored at 4°C.

About 0.5 ml PG (or cellulose), extract was mixed with the reaction solution and incubated at 37°C for 1 h. About 1.5 ml 3,5-dinitrosalicylic acid reagent was immediately added dropwise, heated in a boiling water bath for 5 min, diluted to 25 ml, and was shaken. The absorbance was determined at 540 nm. The enzyme extract was boiled for 5 min and was then used as a blank control group.

#### Measurement of Antioxidant System and Phenylalanine Ammonia-Lyase

To extract peroxidase (POD), polyphenol oxidase (PPO), catalase (CAT), superoxide dismutase (SOD), superoxide anion (${O_{2}}^{-}$), H_2_O_2_, malondialdehyde (MDA), and phenylalanine ammonia-lyase (PAL), 5.0 g peach fruit sample was ground in the extraction buffer solution in an ice bath and centrifuged at 4°C and 12,000 × *g* for 30 min (20 min for ${O_{2}}^{-}$, H_2_O_2_, and MDA), and the supernatant was used for further measurement.

Started timing as soon as 0.5 ml POD extract (0.1 ml for PPO extract or CAT extract) was mixed rapidly with 3.0 ml guaiacol (25 mmol/L) and 0.2 ml H_2_O_2_ (4.0 ml 50 mmol/L pH 5.5 sodium acetate-acetic acid buffer solutions and 1.0 ml catechol for PPO, 2.9 ml 20 mmol/L H_2_O_2_ for CAT). The absorbance was measured at 470 nm (420 nm for PPO, 240 nm for CAT), considering distilled water as a reference. The reaction was measured first in 15 s, and it was recorded as the initial value and then recorded at every 1 min (1 min for PPO, 30 s for CAT) and was continuously measured for 6 times (7 times in total).

About 0.1 ml SOD extract and the reaction solution were sequentially added to the measuring tube. The blank control group replaced the supernatant with phosphate buffer solution (0.1 mol/L, pH 7.8). A blank tube was placed in the dark, and the rest were placed under a 4,000 lx fluorescent lamp for 15 min. The reaction was immediately terminated in the dark. The blank tube, which had been placed in the dark, was zero-referenced, and the absorbance value was measured at 560 nm.

About 1.0 ml ${O_{2}}^{-}$ extract was mixed with 1.0 ml phosphate buffer solution (50 mmol/L, pH 7.8) and 1.0 ml hydroxylamine hydrochloride solution (1 mmol/L) and incubated at 25°C for 1 h. Next, 1.0 ml p-aminobenzenesulfonic acid (17 mmol/L) and 1.0 ml α-naphthylamine (7 mmol/L) were added and incubated at 25°C for 20 min. Eventually, absorbance was immediately measured at 530 nm. The mixture without incubation for 1 h was used as a blank control group.

About 1.0 ml H_2_O_2_ extract was mixed with 0.1 ml titanium tetrachloride-hydrochloric acid (10%) and 0.2 ml concentrated ammonia and was centrifuged at 4°C and 12,000 × *g* for 15 min after reacting for 5 min. Next, the precipitate was washed 2–3 times with the precooled acetone. Eventually, 3.0 ml sulfuric acid (2 mol/L) was added to the precipitate until completely dissolved. The absorbance was determined at 412 nm.

About 2.0 ml MDA extract was mixed with 2.0 ml thiobarbituric acid (6.7 g/L), refrigerated, and centrifuged after boiling for 20 min in a boiling water bath. The absorbance was determined at 450, 532, and 600 nm. The blank control group replaced the supernatant with 2.0 ml of TCA.

About 0.5 ml PAL extract was mixed rapidly with the reaction solution and incubated at 37°C for 1 h. Eventually, 0.1 ml hydrochloric acid (6 mol/L) was added to stop the reaction. The absorbance was determined at 290 nm. The enzyme extract was boiled for 5 min and was used as a blank control group.

#### Measuring of Superoxide Anion, Chitinase, and β-1,3-Glucanase

The production rate of superoxide anion and the activities of chitinase (CHI) and β-1,3-glucanase (GLU) were measured by superoxide anion kit, CHI kit, and GLU kit, respectively (Comin Biotechnology, Co., Ltd., Suzhou, CHN).

### Statistical Analysis

All the experiments were repeated twice. SPSS 18.0 software (SPSS Inc., Chicago, IL, USA) was used to conduct statistical analysis (ANOVA). Duncan’s *post hoc* test was applied to compare the mean values. A significance level of *p* < 0.05 was used to determine the significant differences. Mean values with standard deviations were reported. Additionally, Origin Pro 9 (OriginLab Corp., Northampton, MA, USA) was used to produce charts.

## Results

### *In vivo* and *in vitro* Inhibitory Effects of Volatile Organic Compounds on the Growth of *M. fructicola*

**Figure 1:**
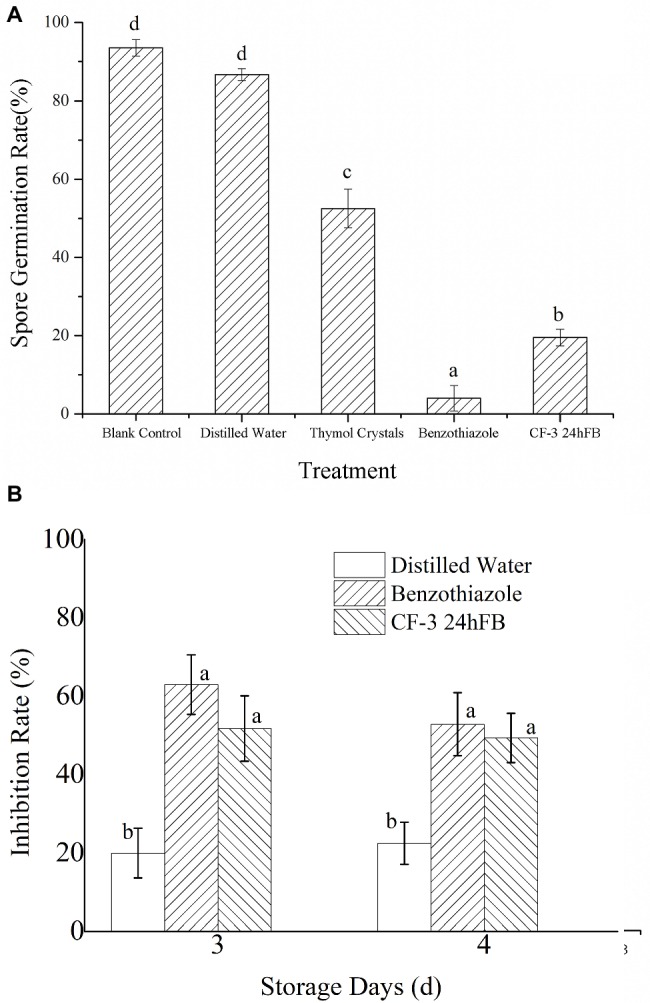
Inhibitory effect of different treatments on spore germination **(A)** and fungus **(B)** of *M. fructicola*. Each column represents the mean value from three independent experiments, and vertical bars represent the standard errors of the means for each treatment. Different letters represent significant differences (*p* < 0.05).

In the spore germination test of *M. fructicola*, 12 h after treatment, spores that were not subjected to any treatment and those treated with distilled water (blank control group) germinated; hence, spore germination rate in all groups was measured at 12 h. What’s more, thymol was used as positive control, due to the fact that thymol fumigation has serious disintegration of membranes and organelles of *M. fructicola* spores and mycelia (Svircev et al., 2007). As shown in Figure 1A, the germination rate following the benzothiazole treatment was 4.01 ± 3.24%, and the germination rate with CF-3 24hFB treatment was 19.59 ± 2.12%.

The antifungal effects of VOCs on *M. fructicola* are presented in Figure 1B. The inhibition rates of benzothiazole and CF-3 24hFB were reported to be nearly 65 and 50% in 3 days, separately.

### *In vitro* Effects of Volatile Organic Compounds on *M. fructicola*

**Figure 2:**
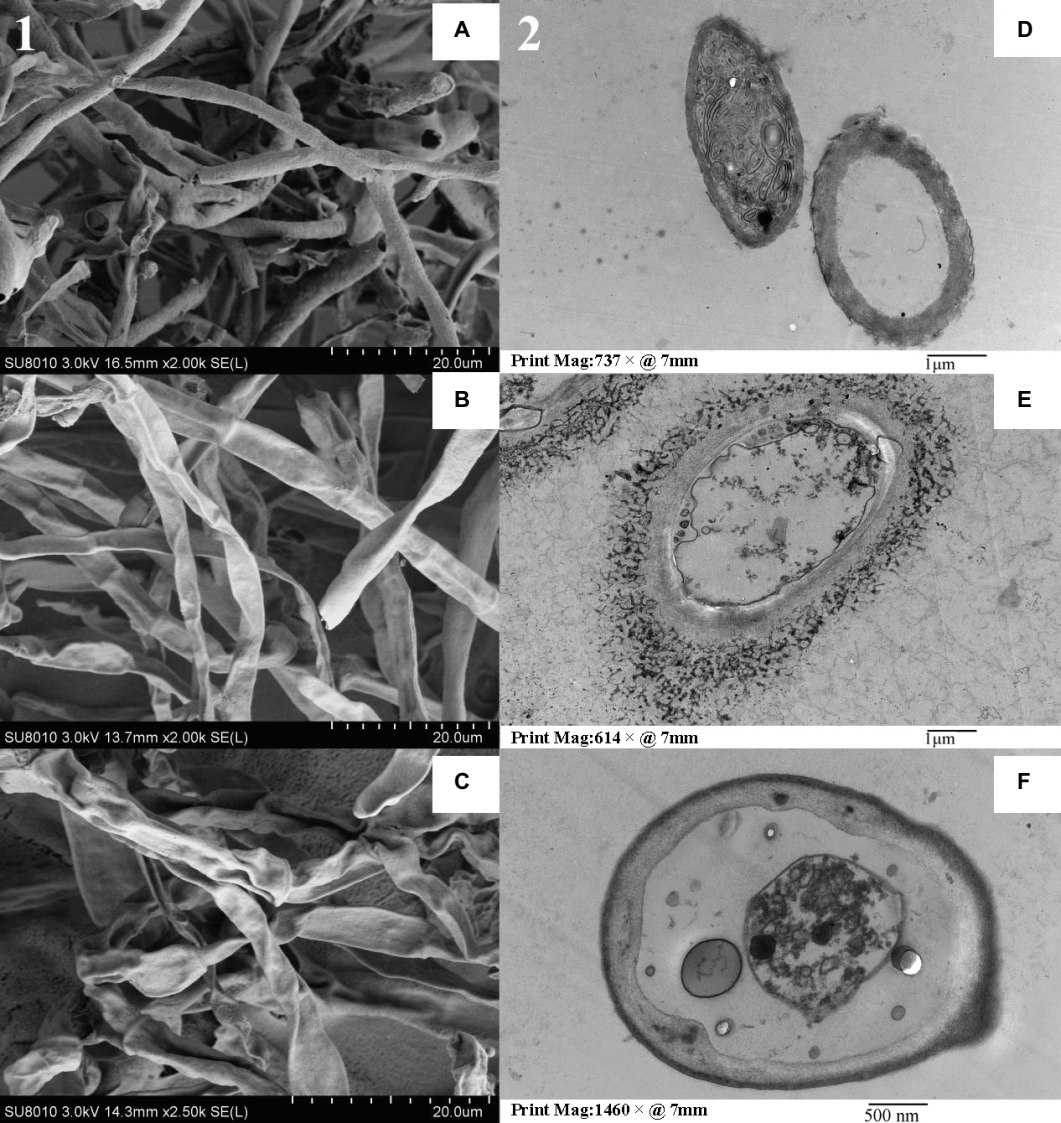
Electron microscope detection for *M. fructicola*. The left side **(1)** is a scanning electron micrograph of *M. fructicola*, and the right side **(2)** is a transmission electron micrograph. **(A)** and **(D)** are blank control, **(B)** and **(E)** are benzothiazole treatment, **(C)** and **(F)** are CF-3 24hFB treatment.

Scanning electron microscopy studies showed that the hyphae in the blank group were intact, and the surface was smooth and full (Figure 2). By contrast, the hyphae shrank, twisted, and ruptured, resulting in a creased or wrinkled surface after benzothiazole and CF-3 24hFB treatment. Transmission electron microscopy showed that the blank group hyphae had typical fungal ultrastructure, normal and uniform cell wall thickness, intact cell membrane, and uniform cytoplasm; however, the treated organelles started to shrink, the plasma membrane ruptured and separated from the cell wall, and the intracellular components were severely destroyed and formed an empty shell, indicating that the VOCs of CF-3 24hFB and benzothiazole can act on the cell wall and cell membrane of the fungus, destroying its barrier function.

The results of GC-MS analysis revealed that *M. fructicola* of the blank control contained more long-chain saturated fatty acids, and the relative content of trans-linoleic acid, oleic acid, and palmitic acid increased after treatment with benzothiazole and CF-3 24hFB (Table 1), indicating that the unsaturation in the cell membrane of *M. fructicola* decreased, and the fluidity of the membrane gradually weakened, resulting in leakage of contents.

**Table 1:** Fatty acid content of cell wall of *M. fructicola.*

Fatty acid	Treatment		
	No treatment	Benzothiazole	CF-3 24hFB
Myristic acid C14:0 (%)	Not detected b	4.53 ± 0.38 a	4.47 ± 0.89 a
Palmitic acid C16:0 (%)	32.46 ± 2.53 a	32.58 ± 4.23 a	34.22 ± 2.98 a
Stearic acid C18:0 (%)	19.80 ± 0.94 b	20.51 ± 2.94 b	27.09 ± 1.25 a
9-Hexadecenoic acid C16:1 (%)	28.60 ± 3.21 a	Not Detected b	Not Detected b
Trans-linoleic acid C18:2 (%)	19.14 ± 1.83 b	30.76 ± 3.02 a	20.67 ± 3.77 b
8,11,14-Docosic acid C22:3 (%)	Not detected b	11.62 ± 2.11 a	13.55 ± 1.78 a
Unsaturated/saturated	0.91 ± 0.02 a	0.74 ± 0.03 b	0.52 ± 0.02 c

*The mean and standard error in the table were taken from three replicates, and the different letters indicated that various treatments of the same strain differed significantly (p < 0.05)*.

The ergosterol content in the cell wall of *M. fructicola* treated with benzothiazole and CF-3 24hFB significantly reduced (Table 2), indicating that the treatment with VOCs can reduce ergosterol content in the cell wall, affect the integrity of the fungal cell wall, and thus weaken the cell membrane and material transport, thereby affecting the viability of fungal cells.

**Table 2:** Content of ergosterol in *M. fructicola.*

	Treatment	Peak area ratio	Ergosterol content (mg/g)
*Monilinia fructicola*	No treatment	0.7658 ± 0.0122	0.2448 ± 0.0071 a
	Benzothiazole	0.5933 ± 0.0325	0.1442 ± 0.0190 b
	CF-3 24hFB	0.5053 ± 0.0235	0.0928 ± 0.0137 b

*The mean and standard error in the table were taken from three replicates, and the different letters indicated that various treatments of the same strain differed significantly (p < 0.05)*.

### *In vivo* Effects of CF-3 Volatile Organic Compounds on Peach

**Figure 3:**
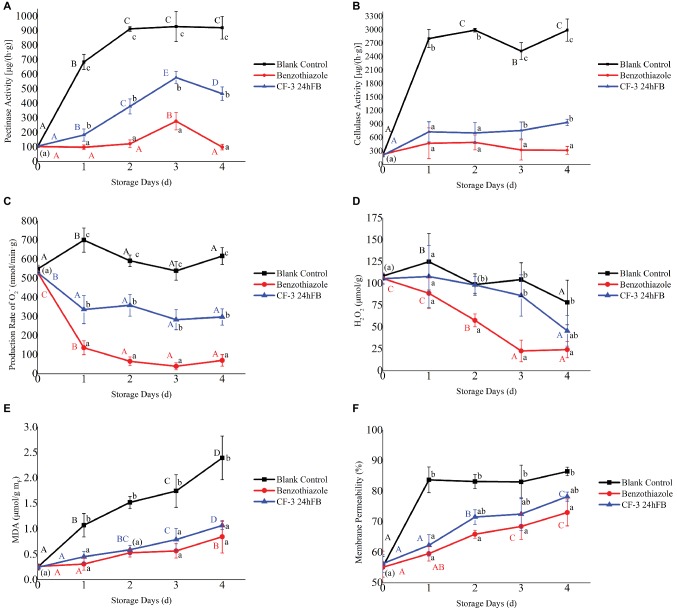
Influence of different treatments on pectinase **(A)**, cellulose **(B)** activity, the production rate of superoxide anion **(C)**, amount of hydrogen peroxide **(D)**, amount of MDA **(E)**, and membrane permeability **(F)** *in vivo* during storage. The mean and standard error in the figure were taken from three replicates, and the different lowercase letters indicated that various treatments during the same storage days differed significantly different, the different uppercase letters indicated that same treatment during different storage days differed significantly different (*p* < 0.05).

As can be observed from Figure 3, the polygalacturonase activity in the peach fruit of the blank group and the CF-3 24hFB treatment group increased conspicuously in 3 days then fell back (Figure 3A); however, the magnitude of increase and the enzyme activity in the blank group were much greater than those in the CF-3 24hFB treatment group. And the enzyme activity of the benzothiazole treatment group was lower than that of the other two groups. The cellulase level in peach fruit increased rapidly as the number of storage days increased, whereas the two treatment groups were not obviously different in 2 days, and the activity in the benzothiazole treatment group was the lowest in 3 and 4 days (Figure 3B).

The production rate of superoxide anion and hydrogen peroxide content in peach fruit during storage is presented in Figures 3C,D. During the whole storage process, except for the blank control group, the production rate of superoxide anion in the other two groups fluctuated obviously. The production rate of superoxide anion in the benzothiazole treatment group was overall significantly lower than that in the other groups. Moreover, the general trend of hydrogen peroxide content was decreasing. Except for the benzothiazole treatment group, no significant difference was observed in the hydrogen peroxide content between the other two groups, whereas the hydrogen peroxide content in the benzothiazole treatment group was significantly lower than that in the blank control group.

The changes in MDA content and cell membrane permeability during storage are presented in Figures 3E,F. During the whole storage process, the MDA content in the peach fruit of the two treatment groups increased with storage time, but there was no significant difference between the two groups; however, the MDA content significantly differed from that of the blank control group. During the storage process, cell membrane permeability increased significantly, indicating that the damage of fruit cells became much more serious as the storage days increase, but there was no significant difference in cell permeability between the two treatment groups, and the difference was significantly lower than that in the blank control group. Among these, the MDA content and cell membrane permeability were the lowest in the benzothiazole treatment group.

**Figure 4:**
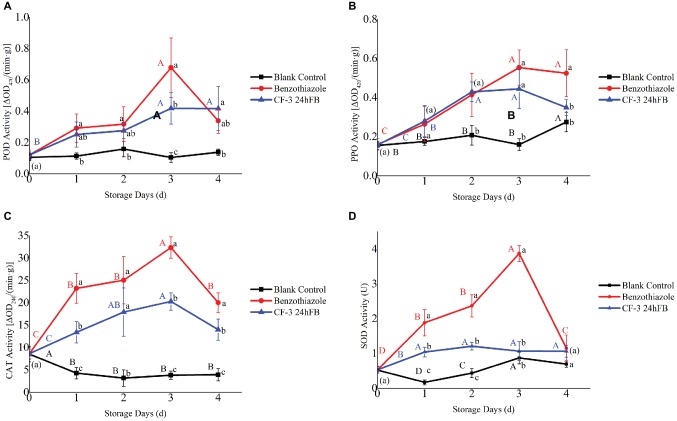
Influence of different treatments on POD **(A)**, PPO **(B)**, CAT **(C)**, and SOD **(D)** activity *in vivo* during storage. The mean and standard error in the figure were taken from three replicates, and the different lowercase letters indicated that various treatments during the same storage days differed significantly different, the different uppercase letters indicated that same treatment during different storage days differed significantly different (*p* < 0.05).

The activities of four antioxidant-related enzymes (PPO, POD, CAT, and SOD) initially revealed an increasing trend and then decreased during storage (Figure 4). Enzymatic activities in treated peaches were higher than those in the blank control group. Among these, the activities of the four antioxidant enzymes in the benzothiazole treatment group were higher than those in the CF-3 24hFB treatment group, especially in 3 and 4 days, and both peaked on the third day.

**Figure 5:**
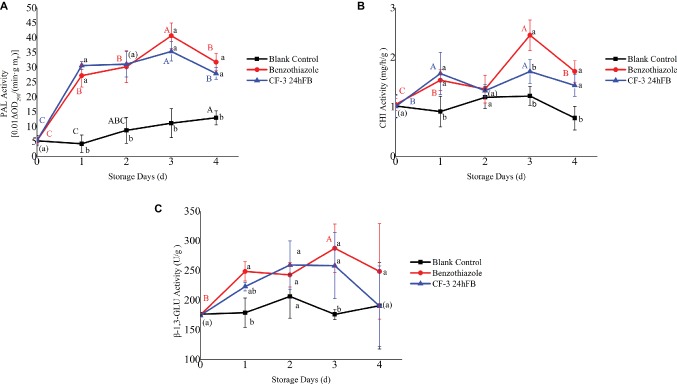
Influence of different treatments on PAL **(A)**, CHI **(B)**, and β-1,3-GLU **(C)** activity *in vivo* during storage. The mean and standard error in the figure were taken from three replicates, and the different lowercase letters indicated that various treatments during the same storage days differed significantly different, the different uppercase letters indicated that same treatment during different storage days differed significantly different (*p* < 0.05).

The activities of three enzymes related to disease resistance in plant (PAL, CHI, β-1,3-GLU) initially revealed an increasing trend and then decreased during storage (Figure 5), and after two treatments, the activities of the three enzymes in the peaches were all higher than those of the blank control group as a whole. Among these, the activities of the three disease-resistant enzymes in the benzothiazole treatment group were higher than those in the CF-3 24hFB, and both peaked on the third day.

## Discussion

We previously reported by using both physical and flat-panel experiments that VOCs produced by *B. subtilis* CF-3 can prevent the decay of peaches and significantly inhibit the growth of pathogenic fungi as well as spore germination (Gao et al., 2018). It is further indicated that VOCs can destroy fungal cells *in vitro* and induce disease resistance in postharvest peaches.

The inhibition rate indicated that the fruits in benzothiazole and 24hFB treatment groups were much better than those in the sterile water and blank groups. Namely, CF-3 VOCs can really prevent decay. *In vitro*, the lipid composition of the fungal cell membrane and its content are important parameters of the cell membrane, which have several important functions, including increasing cell membrane stability, regulating cell membrane fluidity, and reducing the permeability of water-soluble substances (Heaton and Randall, 2011). If the saturated fatty acid in the cell membrane is at a high level, it will lead to an increase in the cell membrane stiffness, causing cell rupture (Bayer et al., 2000). In this experiment, the unsaturation of fungal cells treated by VOCs decreased to varying degrees, which could lead to the decrease of membrane fluidity, causing cell rupture and leakage of contents as reported (Cronan, 2003). In addition, ergosterol is a major component of the cell wall of filamentous fungi and is helpful for the fluidity and asymmetry and integrity of cell membranes, thus playing an essential role in the growth and function of cells (Lupetti et al., 2002). In this experiment, the content of ergosterol in the wall of fungal cells decreased after treated by VOCs, which could reflect that VOCs destroyed the cell wall of the fungus and interfered with the normal operation of the cell membrane.

By studying the effects of VOCs produced by *B. subtilis* CF-3 on the antioxidant system and disease-resistant enzymes *in vivo*, the effect of VOCs inducing peach fruit resistance to *M. fructicola* was explored. Initially, pectinases play significant roles in the firmness and cell structure (Prade et al., 1999), and cellulase degrades the cellulose and the β-1,4-glucan backbone of xyloglucan (Sethu Priya et al., 1996), which are two important pathogenic enzymes for postharvest harvesting of fruits and vegetables. The pectinase and cellulase activities in peach fruit were significantly lower than those in the blank control group after VOCs fumigation, explaining that the structural damage was alleviated. Moreover, the production rate of superoxide anion and hydrogen peroxide content, two main oxygen-derived species, was significantly lower than the blank control group, indicating that the excessive peroxide production was effectively inhibited. As the final product of lipid peroxidation, MDA is often used as an indicator of cellular oxidative damage under stress (Lu et al., 2014). MDA content and cell membrane permeability in the treatment groups were significantly lower than the blank control group, proving that the oxidative damage of plant cell membrane was smaller. By contrast, the enzyme activities of the four antioxidant enzymes (POD, PPO, CAT, and SOD) were significantly increased. It is reported that benzo-(1,2,3)-thiadiazole-7-carbothioic acid S-methyl ester (BTH) can increase the activity of defense enzymes, including PPO, POD, and CAT in strawberries, enhancing the biocontrol efficacy (Zhang et al., 2015a,b), which provides evidence that the peach fruit treated by VOCs can activate defense enzymes in 3 day, and combined ROS index, it can be seen that VOCs effectively produce antioxidant activity to prevent oxidative damage of plant cells. Eventually, CHI and β-1,3-GLU can catalyze the hydrolysis of chitin and β-1,3-glucan on fungal cell walls (Meng et al., 2010). The previously reported studies revealed that 2,3-butanediol treatment could significantly increase the activity of PAL and CHI and induce *Agrostis stolonifera* for resistance against *Rhizoctonia solani* (Shi et al., 2018). In our study, the activity changes of the three pathogenic enzymes (PAL, CHI, and β-1,3-GLU) are similar to the antioxidant enzymes, which can act on the fungal cell well and effectively prevent infection by fungi.

In summary, the main results of this study are as follows: *in vitro*, CF-3 VOCs can inhibit spore germination of pathogenic fungi, cause abnormalities in cell morphology, and decrease cell membrane fluidity and cell wall integrity. *In vivo*, VOCs can reduce enzyme activities of pathogenic fungi that are involved in decomposition of plant tissues, activate antioxidant enzymes in fruits to remove excess ROS, thus preventing plant cell damage, and activate disease-resistant enzymes to prevent the invasion of pathogenic fungi, thereby inducing resistance to the fungi. CF-3 VOCs reduce fruit decay, and we may establish a foundation for broad applications in production.

## Data Availability

The raw data supporting the conclusions of this manuscript will be made available by the authors, without undue reservation, to any qualified researcher.

## Author Contributions

HG, PL, and MZ designed the experiments. PL, MZ, and PZ performed the experiments. PL, MZ, SW, and PZ analyzed the data. HG, MZ, PL, and SW drafted the manuscript. All authors read and approved the final manuscript.

### Conflict of Interest Statement

The authors declare that the research was conducted in the absence of any commercial or financial relationships that could be construed as a potential conflict of interest.
